# Targeted Inhibition of CHD1L by OTI-611 Reprograms Chemotherapy and Targeted Therapy-Induced Cell Cycle Arrest and Suppresses Proliferation to Produce Synergistic Antitumor Effects in Breast and Colorectal Cancer

**DOI:** 10.3390/cells14050318

**Published:** 2025-02-20

**Authors:** Hector Esquer, Qiong Zhou, Daniel V. LaBarbera

**Affiliations:** 1 Department of Pharmaceutical Sciences, The Skaggs School of Pharmacy and Pharmaceutical Sciences, The University of Colorado Anschutz Medical Campus, Aurora, CO 80045, USA; hector.esquer@cuanschutz.edu (H.E.); qiong.zhou@cuanschutz.edu (Q.Z.); 2 The Center for Drug Discovery, The University of Colorado Anschutz Medical Campus, Aurora, CO 80045, USA; 3 The University of Colorado Cancer Center, Aurora, CO 80045, USA

**Keywords:** CHD1L, CHD1L inhibitor (CHD1Li), combination therapy, cell cycle, Cyclin D1, G1 arrest, cancer, antitumor mechanism of action, breast cancer, colorectal cancer

## Abstract

The second and third most frequently diagnosed cancers worldwide are breast (2.3 million new cases) and colorectal (1.9 million new cases), respectively. Although advances in cancer therapies and early detection have improved the overall survival of patients, patients still develop resistance or cancer recurrence. Thus, the development of novel therapies that can affect multiple mechanisms of drug resistance and cell survival is ideal for the treatment of advanced and metastatic cancers. CHD1L is a novel oncogenic protein involved in regulating chromatin remodeling, DNA damage repair, epithelial-mesenchymal transition (EMT), and programmed cell death via PARthanatos. Herein, we assess in real-time how the CHD1L inhibitor (CHD1Li) OTI-611 modulates cell cycle progression in Colo678, SUM149PT, and SW620 cell lines. By utilizing a cell cycle reporter, we tracked the real-time cell cycle progression of cancer cells treated with OTI-611 alone and in combination with standard-of-care (SOC) therapies. Our results indicate that OTI-611 causes G1 phase cell cycle arrest through a CHD1L-mediated mechanism that regulates Cyclin D1 expression and localization. As a result of this mechanism, OTI-611 can reprogram the cell cycle effects of other antitumor agents to modulate and arrest cells in G1 when used in combination, including agents commonly known to arrest cells in the G2/M phase. Therefore, we conclude that OTI-611-induced G1 arrest represents a critical component of its unique mechanism of action, contributing significantly to its anticancer activity.

## 1. Introduction

The global burden of cancer is projected to rise to 35 million new cases annually by 2050 [1]. As of 2022, GLOBOCAN reported approximately 20 million new cancer cases worldwide, with breast cancer (BC) and colorectal cancer (CRC) being the second and third most frequently diagnosed cancers, accounting for 2.3 million and 1.9 million new cases, respectively. In the United States, the prognosis for metastatic BC and CRC patients remains poor, with 5-year survival rates of approximately 27% and 13%, respectively [2]. Standard-of-care (SOC) treatments, including chemotherapy, hormone therapy, and targeted therapies, often fall short due to low patient responses and the pervasive challenges of tumor progression, metastasis, and multi-drug resistance (MDR). Targeting Chromodomain Helicase DNA-Binding-Protein 1-Like (CHD1L), also known as Amplified in Liver Cancer-1 (ALC1), offers a promising strategy to combat these challenges and improve survival outcomes for cancer patients.

A growing body of evidence links CHD1L as an oncogenic protein and therapeutic target in a multitude of cancers [3,4,5,6,7,8,9,10,11,12,13,14]. CHD1L plays a pivotal role in driving tumor progression, metastasis, and MDR through its unique chromatin remodeling activity [6,7,8]. CHD1L is unique from other chromatin remodelers in that it possesses a C-terminal macro domain, which may render the enzyme autoinhibited in many cell types. However, in highly proliferating cells such as cancer, CHD1L is activated through poly-ADP-ribose (PAR) binding to CHD1L’s macro domain [15,16,17,18]. Once activated, CHD1L regulates numerous oncogenic functions, including malignant gene expression, tumor cell plasticity via epithelial–mesenchymal transition (EMT), and cancer stem-cell stemness [3,5,6,7,8]. However, a key oncogenic function of CHD1L appears to be its role as a master regulator of tumor cell survival by controlling the DNA damage response (DDR), cell cycle, and proliferation; and inhibiting programmed cell death mechanisms [6,7,8,19,20,21,22,23,24]. Clinical studies and analyses of patient samples show that elevated CHD1L expression is correlated with poor prognosis and increased metastatic potential across many cancer types [3,8,13,25,26,27,28].

Our group reported the discovery and optimization of the first small-molecule inhibitors of CHD1L (CHD1Li) [3,5]. CHD1Li OTI-611 represents a promising lead drug that displays excellent antitumor activity alone or in combination with SOC chemotherapy and targeted therapy both in vitro and in vivo against BC and CRC [5,6,7]. Targeted allosteric inhibition of CHD1L by OTI-611 impairs its nucleosome remodeling function by trapping CHD1L onto chromatin [6,7]. Furthermore, OTI-611-mediated CHD1L inhibition also traps poly(ADP-ribose) polymerase 1/2 (PARP1/2) on chromatin, leaving PAR chains unprotected and open for hydrolysis, which leads to the induction of PARthanatos programmed cell death [6,7].

In this study, we focus our attention on the cell cycle and proliferation in BC and CRC cells. The cell cycle is a tightly regulated process that cancer cells exploit to increase proliferation and evade replication stress and DNA damage [29,30]. Matthews et al. described how cancer cells bypass cell cycle exit checkpoints to sustain continuous division [24]. The cell cycle consists of two main stages: interphase, where the cell duplicates its content, and the mitotic phase (M phase), where the cell divides into two identical daughter cells. DNA replication occurs during the synthesis phase (S phase) of interphase, flanked by two gap phases: G1 (before S phase) and G2 (after S phase). Cancer cells exploit these stages to proliferate uncontrollably, driving tumor progression [30]. Targeting these mechanisms has led to the development of cell cycle inhibitors and modulators, many of which are in clinical trials or approved for combination therapy [29,30,31,32]. Therefore, the goal of this study was to explore and characterize CHD1L’s role in regulating the cell cycle and the consequences of pharmacological inhibition by OTI-611.

CHD1L expression has been linked to enhanced G1/S phase transition and reduced apoptosis in multiple cancers [13,21,33]. Here, we evaluated the effects of OTI-611, chemotherapy, and the PARP inhibitor (PARPi) Olaparib in BC and CRC cells, both as single agents and in combination, using high-content imaging (HCI) real-time cell cycle analysis. Our findings demonstrate that OTI-611 alone induces G1 cell cycle arrest, downregulates Cyclin D1, and inhibits cancer cell proliferation more effectively than chemotherapy or PARPi alone. Additionally, OTI-611 synergizes with chemotherapy to enhance G1 arrest. Furthermore, our results show that inhibiting CHD1L with OTI-611 can reprogram chemo/targeted therapy-induced G2/M arrest into G1 arrest in a dose-dependent manner, disrupting cell cycle progression and proliferation to kill tumor cells.

Our recent seminal work [6,7], along with the findings presented herein, highlights the unique mechanism of action (MOA) of CHD1Li OTI-611. This promising therapeutic strategy inhibits tumor progression, overcomes MDR, and enhances tumor cell death, particularly when used in combination with chemotherapy or other targeted therapies.

## 2. Materials and Methods

### 2.1. Reagents and Antibodies

Etoposide (Cat. No. 80055-248, Millipore-Sigma, Burlington, MA, USA); SN-38 (Cat. No. S4908, Selleckchem, Houston, TX, USA); 5-Fluorouracil (Cat. No. S1209, Selleckchem); mouse anti-Cyclin D1 monoclonal primary antibody (Cat. No. AHF0082, ThermoFisher, Waltham, MA, USA); rabbit anti-Ki-67 monoclonal primary antibody (Cat. No. 9129, Cell Signaling Technology, Danvers, MA, USA); mouse anti-CDK4 monoclonal primary antibody (Cat. No. 2906, Cell Signaling Technology); anti-mouse Alexa Fluor Plus 488 polyclonal secondary antibody (Cat. No. A32723, ThermoFisher); anti-rabbit Alexa Fluor Plus 647 polyclonal secondary antibody (Cat. No. A32733, ThermoFisher); Incucyte^®^ Cell Cycle Green/Red Lentivirus Reagent (Cat. No. 4779, Sartorius, Arvada, CO, USA); and Hoechst 33342 (Cat. No. H3570, ThermoFisher).

### 2.2. Cell Culture

Colo678 (CRC), SUM149PT (BC), and SW620 (CRC) cells were obtained from ATCC or CU Anschutz Cell Technologies Shared Resource. Cell lines were short-tandem repeat (STR)-profiled and were tested for mycoplasma contamination before use. Colo678 and SW620 cells were cultured in RPMI-1640 medium (Cat. No. 11875-093, Gibco, Waltham, MA, USA) supplemented with 10% fetal bovine serum (FBS Cat. No. 10437028, Gibco). SUM149PT cells were cultured in Ham’s F12/Glutamax medium (Gibco) supplemented with 5% FBS, 10 mM HEPES, 1 µg/mL hydrocortisone, and 5 µg/mL insulin. Cells were sub-cultured in 100 mm cell culture dishes (Cat. No. 12-556-002, ThermoFisher).

### 2.3. Lentivirus Transduction

Cells were seeded in growth media 24 h before viral transduction. Incucyte^®^ Cell Cycle Red/Green lentivirus reagent was added to ~30% confluent cells at MOI = 3TU/cell with the presence of 8 µg/mL of polybrene. Media were exchanged after 24 h, and cells were expanded, selected with puromycin, frozen back, and used for experiments.

### 2.4. Drug Treatment Concentrations

A range of optimal drug concentrations were selected for OTI-611 based on our previous studies on the role of CHD1Li in promoting PARthanatos, inhibition of DNA damage response, and its synergistic effects in combination with chemotherapy [6,7]. These doses were then adjusted per cell line and per experiment to a range of (0.5–5 µM). Likewise, a range of chemotherapy doses was selected based on our previous studies for Etoposide [3], SN-38, 5-FU [6], and Olaparib [7].

### 2.5. Real-Time Cell Cycle Measurements

SUM149PT (10,000 cells/well) and SW620 (15,000 cells/well) cells were seeded into 96-well Greiner µClear^®^ Black Tissue Culture Plates (Cat. No. 655090, Monroe, NC, USA) for 24 h, while Colo678 (15,000 cells/well) cells were allowed to attach for 72 h in 100 µL of their corresponding media. Afterward, media were replenished; brought to a final volume of 200 µL; and then cells were treated in a dose–response manner using the FlexDrop™ non-contact dispenser (Revvity), with droplet detection enabled, for single drug treatments and combinations. The plate was placed on an orbital shaker at 200 RPM for 5 min and then imaged using the Incucyte^®^ S3 (Sartorius) outfitted with the S3/SX1 G/R module for image acquisition at 10× every 2 h for 72 h in a 37 °C, humidified, 5% CO_2_ cell incubator. Cell analysis was performed using the Incucyte^®^ Software version 2022B Rev2. Briefly, the analysis settings were the following: Phase Segmentation (AI Confluence), Green and Red Cell Segmentation (Surface Fit), Threshold (GCU) = 2.0, Edge Split On, and Spectral Unmixing (% R contributes to G = 8; % G contributes to R = 0). Cell cycle categorization was performed as recommended by Sartorius’ Cell Cycle Reagent protocol based on green and red fluorescence. Two images were acquired per well. Cell cycle populations were analyzed and presented in percentages in the following recommended distributions: red cells (G1 phase), green cells (G2/M phase), and yellow cells (G1/S phase).

### 2.6. Cyclin D1, Ki-67, and CDK4 Immunofluorescence

Colo678 cells were seeded into Phenoplate™ 96-well microplates (Cat No. 6055302, Revvity) and allowed to attach for 72 h in 100 µL total volume of media. Media were then gently aspirated and exchanged to a final volume of 200 µL. Cells were then treated for 24 h using the FlexDrop™ non-contact dispenser (Revvity). After treatment, media were gently aspirated and replaced with 40 µL of 4% Paraformaldehyde (Cat. No. 15714-S, Electron Microscope Sciences, Hatfield, PA, USA) diluted in 1X PBS and incubated at room temperature for 15 min. Cells were then promptly washed twice with 140 µL of Phosphate-Buffered Solution (PBS), incubated with 30 µL of Blocking Buffer (1X PBS/5% Bovine Serum Albumin (BSA)/0.3% Triton™ X-100), and placed on an orbital shaker at 200 RPM for 1 h. Afterward, the Blocking Buffer was removed, and cells were incubated overnight (4 °C) on an orbital shaker at 200 RPM with mouse anti-Cyclin D1 primary antibody (1:500), rabbit anti-Ki-67 primary antibody (1:1000), or mouse anti-CDK4 primary antibody in 30 µL of Antibody Dilution Buffer (1X PBS/1% BSA/0.3% Triton™ X-100). Next, cells were washed twice with 1X PBS and then incubated with secondary antibody (Alexa Fluor 488 or 647 (1:500), ThermoFisher) at room temperature on an orbital shaker at 200 RPM for 1 h. Finally, cells were washed twice with 1X PBS and incubated with Hoechst 33342 (2.5 µg/mL) for 10 min prior to imaging on the Opera Phenix Plus High-content Screening system (Revvity) across 49 fields of view with a 40× water objective (NA 1.1). Cellular compartments were segmented, analyzed, and quantified using Harmony 5.2 software (Revvity).

### 2.7. Statistical Analysis

Statistical analysis was performed using GraphPad Prism 10 (GraphPad Software, San Diego, CA, USA). All experiments were conducted as two independent experimental replicates, unless otherwise noted, and each experimental condition was performed in triplicates. Data are shown as SD or SEM and are noted in the figure legends. Statistical significance is defined as follows: ns *p* > 0.05, * 0.01 < *p* ≤ 0.05, ** 0.001 < *p* ≤ 0.01, *** 0.0001 < *p* ≤ 0.001, and **** *p* ≤ 0.0001.

## 3. Results

### 3.1. CHD1L Inhibition with OTI-611 Disrupts Cyclin D1 Dynamics and Induces G1 Cell Cycle Arrest in Colo678 Cells

Among its oncogenic roles, CHD1L drives tumor progression and enhances tumor cell survival by promoting cell proliferation through the G1/S phase of the cell cycle, an effect that has been observed in various cancers, including BC and CRC [13,21,33,34]. However, while previous studies uncovered the link between CHD1L and cell cycle modulation through genetic silencing, this study will investigate CHD1L’s effects on the cell cycle using small-molecule inhibition with OTI-611.

**Figure 1:**
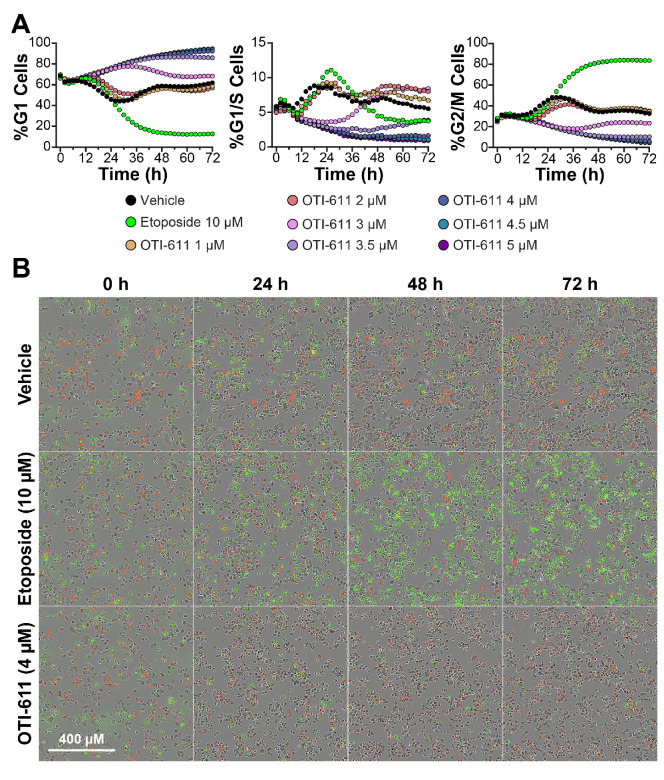
CHD1Li OTI-611 induces G1 cell cycle arrest in CRC Colo678 cells. (**A**) Real-time live cell analysis of cell cycle in Colo678 cells. Data show the mean of two independent experiments in triplicate technical replicates. For each experiment, two fields of view per well were acquired every 2 h. (**B**) Representative phase contrast and fluorescent images of Colo678 cells treated with vehicle (DMSO), etoposide (10 µM), and OTI-611 (4 µM) at different timepoints. Red indicates G1 phase cells, yellow indicates G1/S phase cells, and green indicates G2/M phase cells.

Our recent report indicated that OTI-611 can arrest BC cells in the G2/M phase of the cell cycle [7]. Given the link between CHD1L and G1/S transition, we hypothesized that our previous observations may be influenced by dose and time. To test our hypothesis, we used the Fluorescence Ubiquitin Cell Cycle Indicator (FUCCI) reporter [35] to investigate OTI-611’s effects on the cell cycle in real-time across a range of concentrations, utilizing the Colo678 CRC cell line (Figure 1). At doses between 3.5 and 5 μM, OTI-611 arrested cells in G1 (Video Set S1), a finding which is consistent with reports that show CHD1L is involved in regulating G1/S transition [21,33]. Interestingly, OTI-611 at 3 µM delayed the entry of Colo678 cells into G1/S phase for up to 36 h of treatment. However, after 72 h, the cells resumed progression, resulting in a cell cycle distribution resembling that of the vehicle control. At concentrations below 3 µM, we did not observe G1 cell arrest, aligning with our earlier report of OTI-611 inducing G2/M arrest at sub-micromolar doses [7].

Unlike our previous study using endpoint flow cytometry, the real-time HCI analysis allowed precise identification of OTI-611’s optimal doses for cell cycle disruption. At these doses, OTI-611 effectively arrests cells in the G1 phase, paving the way for a deeper understanding of its mechanistic effects. In contrast, etoposide, as extensively reported [36] and also found on our experiments, arrests cells in the G2/M phase and supports proper FUCCI reporter activity. Collectively, these findings highlight that real-time HCI analysis is a robust and effective method for evaluating tumor cell cycle dynamics, demonstrating its suitability for high-throughput screening applications.

**Figure 2:**
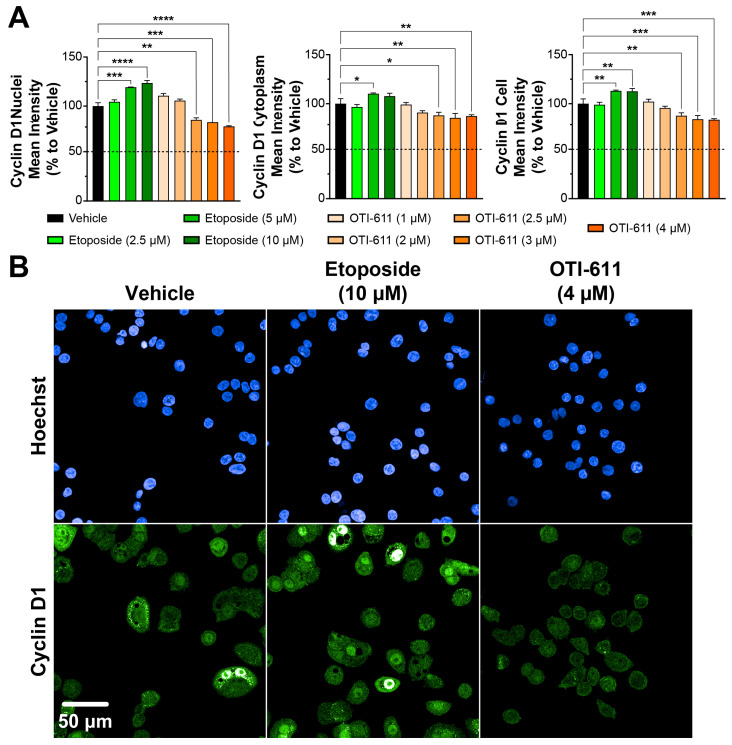
CHD1Li OTI-611 downregulates Cyclin D1 expression and alters its localization in CRC Colo678 cells. (**A**) Fluorescence intensity quantification of Cyclin D1 expression in the nucleus, cytoplasm, and cell (nucleus + cytoplasm) compartments of Colo678 cells. Data were normalized to vehicle (DMSO) and are expressed as the mean ± SD from two independent experiments, each with triplicate wells and 49 acquired fields of view. Significance was calculated using one-way ANOVA (* *p* < 0.05, ** *p* < 0.01, *** *p* < 0.001, and **** *p* < 0.0001). (**B**) Representative immunofluorescence images of Cyclin D1 expression and localization in Colo678 cells. Cells were treated for 24 h and imaged using a 40× water-immersion objective (NA 1.1). Scale bar = 50 µm. Fluorescence signals: Blue = Hoechst 33342 and Green = Cyclin D1.

We measured the effects of OTI-611 and etoposide on G1-to-S-phase transition by examining the nuclear localization of Cyclin D1 and cyclin-dependent kinase-4 (CDK4) via immunofluorescence. Nuclear expression of Cyclin D1 has been recognized to play a leading role in controlling the G1-to-S transition through its interactions with and by activating CDKs, thus promoting cell cycle progression [37,38]. As shown, the treatment with OTI-611 caused a dose-dependent significant downregulation of the nuclear localization of Cyclin D1 (Figure 2A). Immunofluorescence images of Cyclin D1 reveal distinct puncta in the cytoplasm with a uniform nuclear distribution (Figure 2B). Nuclear Cyclin D1 levels are reduced by 25% with a 4 µM concentration of OTI-611 when compared with the control condition. The reduction was significant both in the cytoplasmic compartment (13%) and over the entire cell (16%). The above findings suggest that OTI-611 disrupts Cyclin D1 expression and inhibits G1-to-S progression.

**Figure 3:**
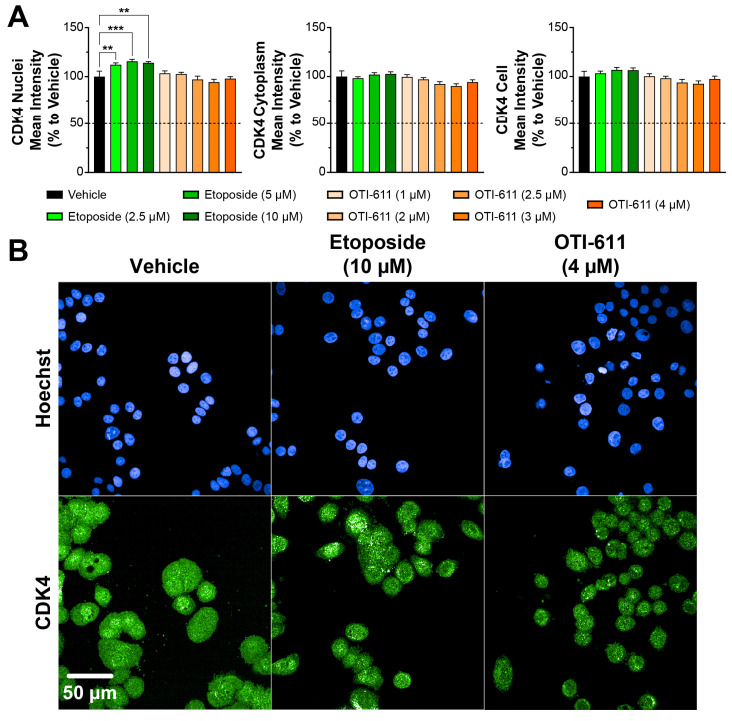
CDK4 localization in Colo678 cells treated with OTI-611 and etoposide. (**A**) Quantitative analysis of CDK4 expression and localization in cells treated with vehicle, etoposide, or OTI-611, as indicated. CDK4 levels in the nucleus, cytoplasm, and total cell were measured as a percentage relative to vehicle. Data are presented as mean ± SD from two independent experiments, each with triplicate wells and 49 acquired fields of view. Statistical significance was calculated using one-way ANOVA (** *p* < 0.01 and *** *p* < 0.001). (**B**) Representative images of immunofluorescence experiments measuring CDK4 expression and localization in Colo678 cells. Cells were treated for 24 h and imaged using a 40× water-immersion objective (NA 1.1). Scale bar = 50 µm. Fluorescence signals: Blue = Hoechst 33342 and Green = CDK4.

Conversely, etoposide increased Cyclin D1 nuclear localization in a dose-dependent manner, likely due to the accumulation of cells transitioning through G1 and S phases but stalled at the G2/M phase, where Cyclin D1 might persist as part of a compensatory response to cell cycle arrest. Etoposide at 5 µM also slightly increased the localization of Cyclin D1 in the cytoplasm (~10%), which may reflect cellular adaptations to DNA damage to maintain homeostasis (Figure 2A). Similar to Cyclin D1, treatment with etoposide caused nuclear accumulation of CDK4; this effect, however, was not significant in the cytoplasmic or whole cell compartments (Figure 3). In contrast, OTI-611 had no significant effects on the expression or cellular localization of CDK4, further highlighting its specific action on Cyclin D1.

**Figure 4:**
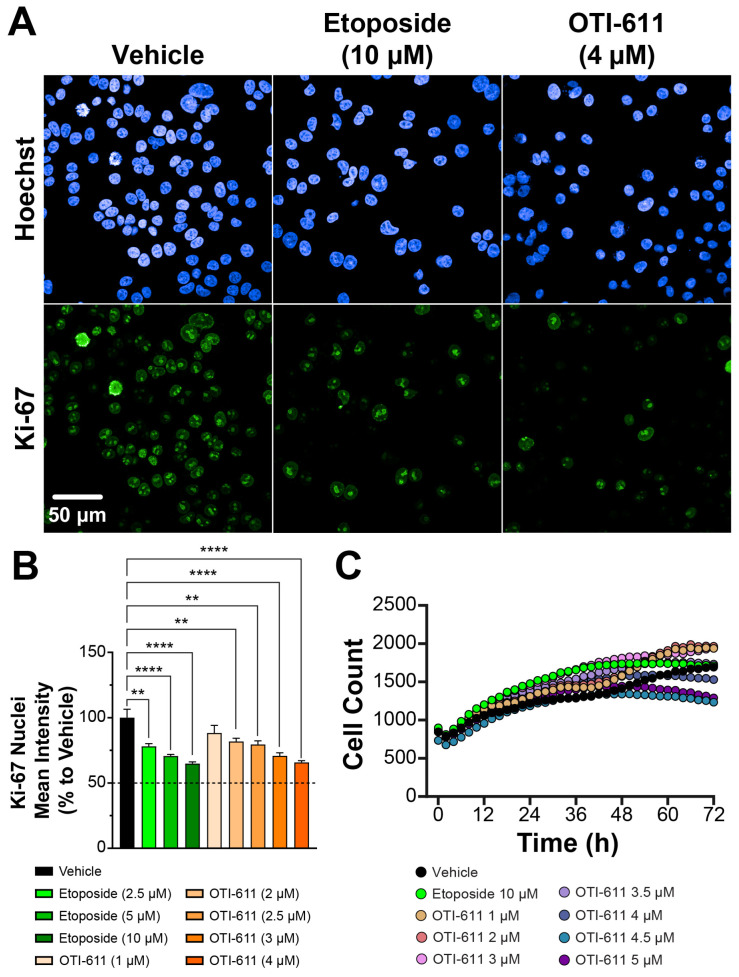
CHD1Li OTI-611 decreases Ki-67 expression in CRC Colo678. (**A**) Representative images of immunofluorescence experiments measuring Ki-67 expression in Colo678 cells. Cells were treated for 24 h and imaged using a 40× water-immersion objective (NA 1.1). Scale bar = 50 µm. (**B**) Fluorescence intensity quantification of Ki-67 expression in the nucleus compartment of Colo678 cells. Data were normalized to vehicle (DMSO) and are expressed as the mean ± SD of duplicate experiments, each performed with triplicate wells and 49 acquired fields of view. Statistical significance was determined using one-way ANOVA (** *p* < 0.01 and **** *p* < 0.0001). (**C**) Time-lapse cell count analysis of Colo678 cells, captured using the Incucyte S3 system. Images were acquired every 2 h at 10× magnification. Data are shown as the mean of two experiments in triplicate technical replicates. Fluorescence signals: Blue = Hoechst 33342 and Green = Ki-67.

Further testing of OTI-611’s broader effects on the cell cycle and its impact on overall proliferation involved studying tumor cell proliferation using Ki-67 immunofluorescent HCI (Figure 4). Ki-67 is a known marker of proliferation during active phases of the cell cycle (G1, S, G2, and M) but is absent during the resting phase (G0) [39]. Treatment with CHD1Li OTI-611 significantly reduced Ki-67 expression in Colo678 cells in a dose-dependent manner over a 24-h period (Figure 4A,B). This reduction indicates a decrease in tumor cell proliferation driven by OTI-611’s disruption of cell cycle progression.

Etoposide also reduced Ki-67 expression, but only at higher concentrations compared to OTI-611. Interestingly, cell counts demonstrated a delayed proliferative effect with higher doses of OTI-611 after 48 h of treatment compared to vehicle (Figure 4C), suggesting potential compensatory mechanisms or secondary effects of CHD1L inhibition on cell cycle re-entry or survival pathways. In contrast, this proliferative delay with etoposide was only observed after 72 h and barely reached levels comparable to vehicle, further underscoring the distinct mechanisms of action between the two treatments.

To the best of our knowledge, this is the first report of etoposide’s ability to induce nuclear accumulation of Cyclin D1 and CDK4, an effect perhaps induced by the accumulation of cells in G2/M phase and compensatory mechanisms of cell growth and cell survival. Furthermore, now in addition to our previously reported MOA of OTI-611 [3,5,6,7], we have uncovered OTI-611’s effects on Cyclin D1 nuclear dynamics, its induction of G1 cell cycle arrest, and its ability to reduce tumor cell proliferation.

### 3.2. OTI-611 Induces G1 Arrest in BC and CRC Cell Lines with Distinct Cell Cycle Dynamics

**Figure 5:**
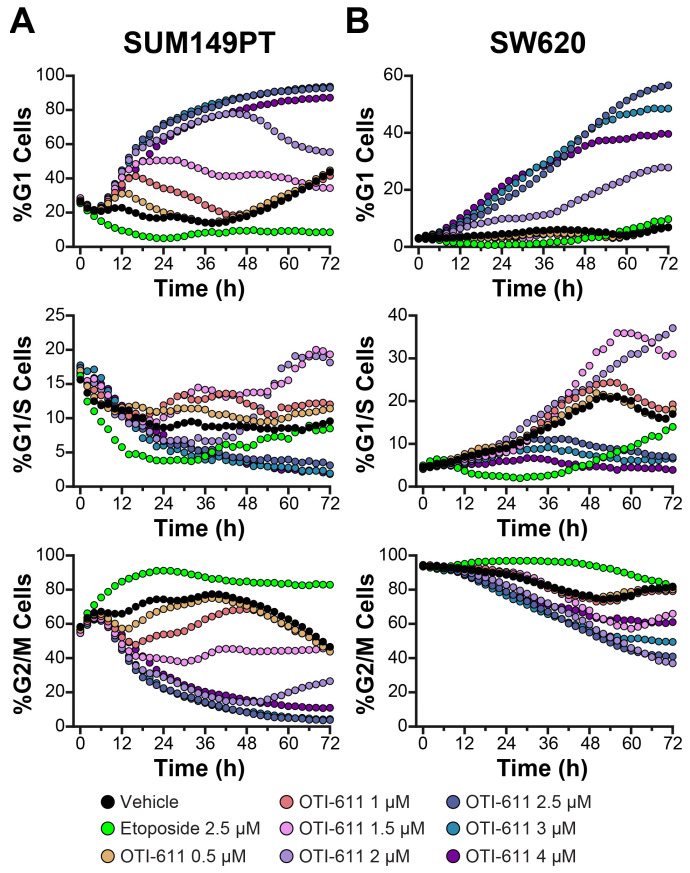
SUM149PT and SW620 cells are arrested in G1 phase when treated with OTI-611. (**A**) Real-time live cell cycle analysis of SUM149PT cells treated with OTI-611. (**B**) Real-time live cell cycle analysis of SW620 cells. The data shown represent the mean of two independent experiments in triplicate technical replicates with two fields of view per well acquired every 2 h.

We examined the effects of OTI-611 on the cell cycle using FUCCI-reporter transduced BC (SUM149PT) and CRC (SW620) cell lines (Figure 5). The response to OTI-611 treatment in SUM149PT and SW620 cells was slower compared to Colo678, likely due to a larger proportion of cells being in the G2/M phase in these lines. However, treatment with OTI-611 in SUM149PT cells led to a time-dependent increase in G1-phase populations, accompanied by a significant reduction in cells in the G2/M phase (Figure 5A and Supplementary Video Set S2). As observed earlier, lower concentrations of OTI-611 (0.5–1.5 µM) induced a transient accumulation of cells in the G1 phase, consistent with prior findings [7]. The extent of this accumulation and the duration of G1-phase occupancy were both dose-dependent.

In contrast to SUM149PT, where a gradual increase in G1-phase populations was observed, nearly all SW620 cells were initially in G2/M phase. However, similar to SUM149PT cells, OTI-611 treatment successfully induced a dose-dependent shift into G1 phase in SW620 (Figure 5B and Supplementary Video Set S3). This effect represents a crucial anticancer mechanism, as OTI-611 treatment shifts SW620 and SUM149PT cells away from pre-mitotic and mitotic populations, highlighting the impact of CHD1L inhibition on cell cycle dynamics. This shift is further corroborated by our cell count data, which demonstrate pronounced sensitivity to OTI-611 treatment, contrasting with the proliferative stalling effect observed with etoposide (Supplementary Figure S1).

### 3.3. CHD1Li OTI-611 Reprograms Etoposide’s G2/M Arrest Toward G1 Arrest in Colo678, SUM149PT, and SW620 Cells

We have reported that CHD1Li OTI-611 strongly synergizes with chemotherapy and other targeted therapy to enhance tumor cell DNA damage and cytotoxicity in BC and CRC cells and tumor organoids [6,7]. In particular, this synergy resulted from OTI-611’s ability to shift chemotherapy cell death mechanisms from apoptosis to PARthanatos. Furthermore, we also showed that OTI-611 can synergize with chemotherapy to induce cell cycle arrest [7]. Thus, we postulated that OTI-611 may synergize with chemotherapy to induce the reprogramming of cell cycle arrest from G2/M to G1. Alternatively, OTI-611 combinations with chemotherapy may induce a mixture of cell cycle arrest phases.

**Figure 6:**
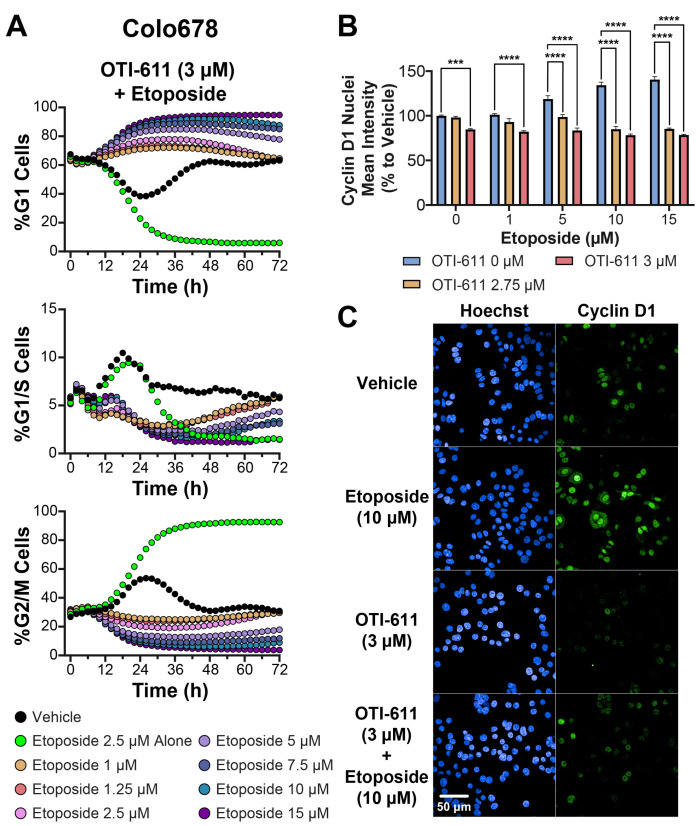
CHD1Li OTI-611 reprograms G2/M cell cycle arrest induced by etoposide to G1 arrest in Colo678 cells. (**A**) Real-time live cell analysis of the cell cycle in Colo678 cells treated with a combination of OTI-611 at 3 µM and Etoposide at various doses. Data are presented as the mean of two independent experiments, each performed in triplicate technical replicates. Two fields of view per well were taken every 2 h. (**B**) Fluorescence intensity quantification of Cyclin D1 expression in the nucleus of Colo678 cells treated for 24 h with OTI-611 and etoposide, alone or in combination. Data were normalized to vehicle (DMSO) and are expressed as the mean ± SEM of duplicate experiments in triplicate wells and 49 fields of view. Significance was calculated using two-way ANOVA (*** *p* < 0.001 and **** *p* < 0.0001) (**C**) Representative images of immunofluorescence experiments measuring Cyclin D1 expression in Colo678 cells. Cells were imaged using a 40× water-immersion objective (NA 1.1). Scale bar = 50 µm. Fluorescence signals: Blue = Hoechst 33342 and Green = Cyclin D1.

We first tested OTI-611 in combination with etoposide. In this experiment, we evaluated OTI-611 at concentrations ranging from 2.5 to 3 µM, while varying etoposide concentrations between 1 and 15 µM in Colo678 cells (Figure 6A and Supplementary Figure S2). Across all conditions, OTI-611 mitigated etoposide-induced G2/M arrest in a dose-dependent manner. Increasing OTI-611 from 2.5 µM to 3 µM progressively enhanced G1 arrest induced when combined with etoposide, achieving complete G1 arrest at 3 µM of OTI-611 (Supplementary Video Set S4). Notably, all tested concentrations of OTI-611 combined with etoposide shifted the percentage of cells in the G1 phase to varying degrees, indicating a strong effect driven by OTI-611.

Furthermore, Cyclin D1 expression and nuclear localization were significantly reduced in a dose-dependent manner when OTI-611 was combined with etoposide (Figure 6B,C). While etoposide alone increased Cyclin D1 accumulation in the nucleus, OTI-611 effectively counteracted this effect, even at high etoposide concentrations. This suggests that OTI-611 exerts a dominant influence on Cyclin D1, reinforcing its role in promoting G1 arrest and disrupting cell cycle progression.

Together, these findings highlight a unique aspect of OTI-611’s MOA, supporting its ability to alter cell cycle dynamics and modulate cell cycle trajectories when used in combination with chemotherapy.

**Figure 7:**
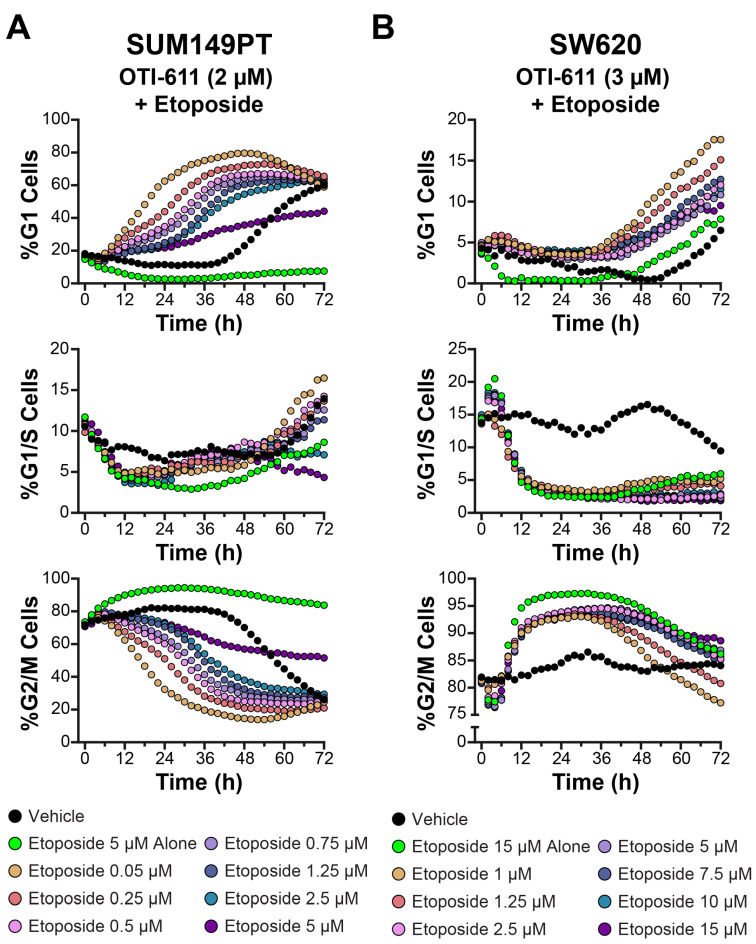
CHD1Li OTI-611 reprograms G2/M cell cycle arrest induced by etoposide to G1 arrest in SUM149PT and SW620 cells. (**A**) Real-time live cell cycle analysis of SUM149PT cells after treatment in combination with OTI-611 and etoposide at various doses. The presented data are the mean of two independent experiments and two images taken per well every 2 h. (**B**) Real-time live cell cycle analysis of SW620 cells treated in combination with OTI-611 and etoposide. The presented data represent the mean of two independent experiments in triplicate technical replicates, with two images captured per well every 2 h.

We further examined the synergistic effects on cell cycle observed between OTI-611 and etoposide combinations using BC (SUM149PT) and CRC (SW620) cell lines (Figure 7 and Supplementary Figures S3 and S4). For these experiments, OTI-611 concentrations were fixed between 1 and 2 µM in SUM149PT and between 2.5 and 3 µM in SW620, while the concentrations of etoposide were varied as indicated. As observed in Colo678 cells, combination treatments of OTI-611 and etoposide produced dose-dependent effects on cell cycle dynamics.

In SUM149PT cells (Figure 7A and Supplementary Figure S3), OTI-611 enhanced G1 arrest in combination with etoposide. Notably, the percentage of cells in the G1 phase increased progressively with rising etoposide concentrations, even when OTI-611 was fixed (Supplementary Video Set S5). This effect was accompanied by a corresponding reduction in G2/M populations, indicating that OTI-611 counteracted etoposide-induced G2/M arrest and redirected cells toward the G1 phase. At higher etoposide concentrations, a synergistic enhancement of G1 arrest was evident, suggesting a cooperative interaction between the two drugs. In SW620 cells (Figure 7B and Supplementary Figure S4), a similar pattern emerged.

OTI-611 consistently mitigated the accumulation of cells in the G2/M phase induced by etoposide. Instead, increasing doses of etoposide, when combined with OTI-611, shifted a significant proportion of cells into the G1 phase. These effects were dose-dependent, with the greatest G1 enrichment observed at the highest etoposide concentrations tested. Additionally, G1/S populations decreased consistently under combination treatments, further supporting the role of OTI-611 in maintaining cells in the G1 phase (Supplementary Video Set S6). These results confirm the MOA of OTI-611 in promoting G1 arrest and counteracting the G2/M accumulation typically induced by etoposide, across multiple cell lines.

### 3.4. Colo678 Cells Are Arrested in G1 Phase in Combination with SN-38 and 5-FU

**Figure 8:**
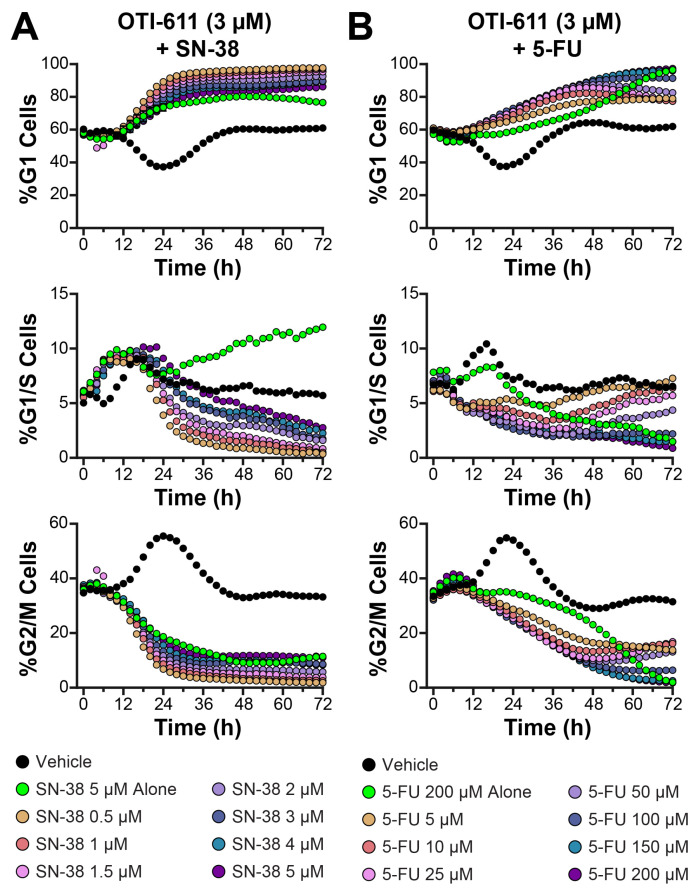
SN-38 and 5-FU synergize with OTI-611 to induce G1 arrest in Colo678 cells. (**A**) Real-time live cell cycle analysis in Colo678 cells after treatment with drug combinations of OTI-611 at 3 µM and SN-38. (**B**) Real-time live cell cycle analysis of Colo678 cells after treatment with OTI-611 at 3 µM and 5-FU. Data are shown as the mean of two independent experiments in triplicate technical replicates, with two images taken per well every 2 h.

We further explored the combination effects of OTI-611 with SOC CRC chemotherapies, SN-38 (the active metabolite of irinotecan), and 5-fluorouracil (5-FU) to build upon our findings with etoposide (Figure 8 and Supplementary Figures S6 and S7). In these experiments, OTI-611 was maintained at concentrations of 2.5–3 µM, while SN-38 and 5-FU were tested across a range of doses. These studies aimed to evaluate OTI-611’s ability to modulate cell cycle dynamics in combination with SOC chemotherapies and to define its unique role compared to etoposide.

SN-38 primarily exerts its cytotoxic effects by stabilizing topoisomerase I-DNA cleavable complexes, leading to DNA strand breaks and cell cycle arrest [40]. Typically, SN-38 induces G2/M arrest; however, it is known to cause G1 arrest in certain cell types [41]. In our experiments, the Colo678 cell line was highly resistant to SN-38, and it predominantly caused G1 arrest instead of G2/M arrest. This unusual behavior may be influenced by the unique genetic background or cell cycle regulation of Colo678 cells, potentially involving p53-related pathways. Prior studies have indicated that the schedule-dependent effects of SN-38 on cell cycle arrest and apoptosis are influenced by p53 functionality [42].

Despite the atypical G1 arrest induced by SN-38 in Colo678 cells, combining it with OTI-611 resulted in a dose-dependent enhancement of G1 arrest across all tested concentrations of SN-38 (Figure 8A and Supplementary Figure S6). These findings suggest that OTI-611 amplifies SN-38’s atypical effects on G1 arrest in Colo678 cells. The literature indicates that SN-38’s capacity to induce G1 arrest becomes more prominent with prolonged exposure or higher concentrations, particularly in cell lines with functional p53 [42]. In resistant Colo678 cells, alternative mechanisms may shift the arrest point to G1, possibly as a compensatory response to DNA damage. This adaptation could restrict progression into S or G2/M phases, further emphasizing the unique interaction between OTI-611 and SN-38 in influencing cell cycle progression.

OTI-611 combined with 5-FU consistently promoted G1 arrest, while reducing G2/M accumulation (Figure 8B and Supplementary Figure S7). Compared to SN-38, the combination with 5-FU resulted in a more gradual increase in the G1 population, suggesting potential differences in the MOA between the two SOC chemotherapies (Supplementary Video Set S8). The observed reduction in G2/M cells and the overall effects on cell cycle dynamics were consistent with those observed with other combination treatments, highlighting the versatility of OTI-611.

Combining OTI-611 with SN-38 significantly reduced cell proliferation compared to vehicle or single-agent treatments (Supplementary Figure S8). This effect was dose-dependent, with higher concentrations of SN-38 leading to the most significant reductions in cell count when combined with OTI-611. These findings further emphasize OTI-611’s potential to enhance the therapeutic efficacy of SOC chemotherapies, partly by modulating cell cycle dynamics and reducing tumor cell proliferation. However, the combination of OTI-611 with 5-FU did not substantially alter cell proliferation, as measured by cell counts after 72 h. This outcome may be attributed to the slow proliferation of Colo678 cells and/or specific resistance mechanisms to 5-FU treatment. Additionally, 5-FU’s anticancer activity partly relies on its ability to inhibit thymidylate synthase and incorporate metabolites into RNA and DNA [43], which could be counteracted if cells efficiently repair these types of DNA damaging mechanisms.

### 3.5. Olaparib-Induced G2/M Arrest in SUM149PT Cells Is Reprogrammed Toward G1 Arrest When Combined with OTI-611

**Figure 9:**
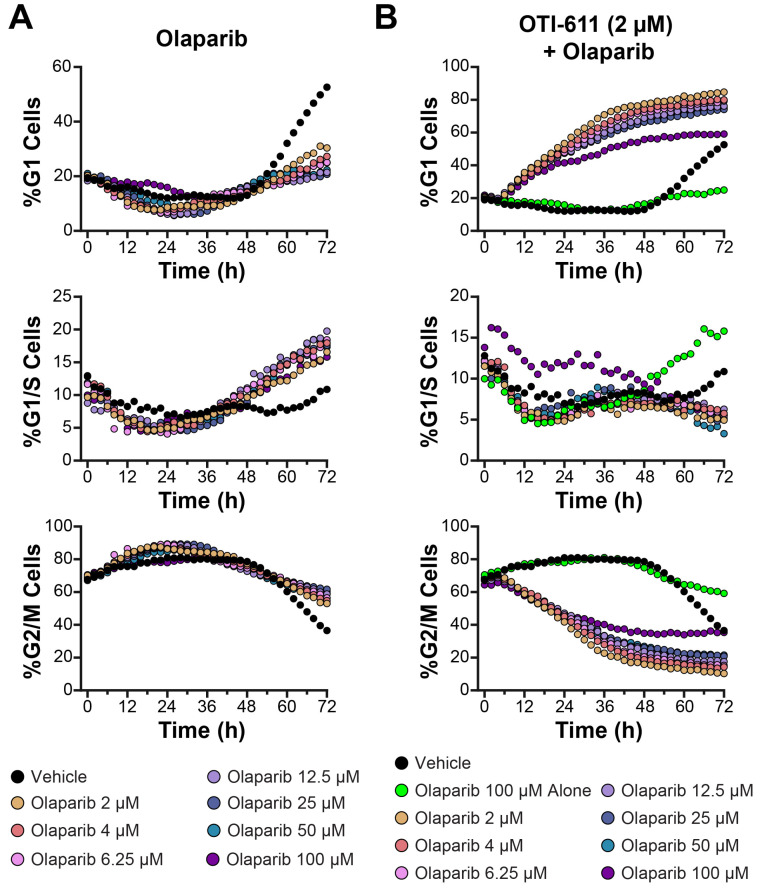
CHD1Li OTI-611 reprograms G2/M cell cycle arrest induced by Olaparib to G1 arrest in SUM149PT cells. (**A**) Real-time live cell cycle analysis of cells treated with Olaparib in a dose–response manner. (**B**) Real-time live cell cycle analysis of cells treated with a combination of OTI-611 at 2 µM and Olaparib over 72 h. Data are shown as the mean of two independent experiments in triplicate technical replicates, with two images taken per well every 2 h.

As with chemotherapy, we previously reported that OTI-611 synergizes with the PARP inhibitor (PARPi) Olaparib to influence various biological processes, including PARPi trapping, DNA damage, and cytotoxicity [7]. To further explore its potential in combination therapy, we studied the effects of OTI-611 combined with Olaparib on the cell cycle in SUM149PT BC cells (Figure 9).

In these experiments, Olaparib was tested across a concentration range of 2–100 µM, both alone and in combination with OTI-611. Treatment with Olaparib alone resulted in modest alterations to the cell cycle, with minimal effects on G1 arrest (Figure 9A and Supplementary Video Set S9). At all tested concentrations, the percentage of cells in the G1 phase remained below 40%, while G2/M populations showed only a moderate decrease over time. These findings suggest that Olaparib alone has a limited impact on G1 arrest in SUM149PT cells, consistent with its primary role in targeting DNA damage repair.

When combined with OTI-611, a dose-dependent enhancement of G1 arrest was observed (Figure 9B and Supplementary Video Set S10). The percentage of cells in the G1 phase increased significantly compared to Olaparib alone, exceeding 60% at higher Olaparib concentrations (≥50 µM). G2/M populations were markedly reduced, indicating that OTI-611 synergizes with Olaparib to promote a robust shift from the G2/M to G1 phase. These effects were accompanied by a consistent reduction in G1/S populations, suggesting an inhibition of cell cycle progression at the G1/S checkpoint. Additional doses and cell counts are shown in Supplementary Figures S9 and S10.

The combination of OTI-611 with Olaparib exemplifies OTI-611’s ability to reprogram the cell cycle effects of other antitumor agents, likely due to its ability to disrupt Cyclin D1. For example, the combination of OTI-611 and Olaparib shifted Olaparib’s typical G2/M arrest to G1 arrest. These results establish OTI-611 as a promising combination partner with PARPi, offering a therapeutic strategy to improve outcomes in cancers dependent on DNA damage repair pathways. Further studies are needed to elucidate the molecular basis of this apparent synergy and assess its applicability to other cancer models.

## 4. Discussion

In this study, our findings support the therapeutic potential of CHD1Li OTI-611 by altering cancer cell cycle dynamics and improving the efficacy of SOC chemotherapy and targeted therapy in BC and CRC cells. Now, in addition to our previously reported MOA of OTI-611 [3,5,6,7], we have uncovered OTI-611’s effects on Cyclin D1 and Ki-67 expression, its induction of G1 cell cycle arrest, and its ability to reduce tumor cell proliferation.

Our understanding of OTI-611-induced cell cycle arrest was facilitated by the development of FUCCI reporter technology. The FUCCI system reports on two critical components of DNA replication control: Cdt1, which peaks during G1 phase, prior to DNA replication, and rapidly declines upon entry into S phase; and Geminin expression, which increases in the S phase and G2 phase, but also rapidly decreases in late mitosis and the G1 phase [35]. Thus, real-time cell cycle analysis by HCI of Cdt1 (red fluorescence) and Geminin (green fluorescence) revealed the time- and dose-dependent nature of OTI-611’s effects on cell cycle progression in Colo678, SUM149PT, and SW620 cells. Furthermore, these findings align with those of other groups, in which CHD1L overexpression promotes G1-to-S phase transition and cancer cell survival [13,21,33]. However, this is the first time that cell cycle modulation and G1 arrest have been shown through pharmacological inhibition of CHD1L enabled by CHD1Li OTI-611.

Moreover, OTI-611-mediated downregulation of Cyclin D1 in the nucleus and cytoplasm supports a plethora of distinct downstream anticancer mechanisms. Cyclin D1 forms complexes with CDK4/6, which then phosphorylates the retinoblastoma protein (Rb), initiating the G1-to-S-phase transition [37]. Cyclin D1 accumulation by mitogenic signaling induces cells to enter the cell cycle. Amplification and overexpression of Cyclin D1 have been observed in numerous cancer types, including BC and CRC [32]. In addition, Cyclin D1 can accumulate in both the nucleus and cytoplasm in tumor cells to induce its oncogenic roles. In the nucleus, Cyclin D1 promotes proliferation, transcription, and the DNA damage response; and controls genomic stability. Meanwhile, in the cytoplasm, Cyclin D1 can regulate cell invasion and migration. Intriguingly, both nuclear and cytoplasmic Cyclin D1 also regulate cell metabolism [37].

Given the tumorigenic functions of Cyclin D1, drugs that can downregulate its expression in both the nucleus and cytoplasm are attractive candidates both alone and in combination with other SOC therapies. Numerous kinase-inhibitor drugs targeting the cell cycle have been approved for clinical use or are in clinical trials but can present limitations via off-target effects and acquired drug resistance [29,30,32]. OTI-611’s lack of significant modulation of CDK4 expression but significant downregulation of Ki-67 and cell proliferation may indicate that it acts early in the cell cycle machinery. This mechanism is supported by Cyclin D1 reduction and the rapid induction of G1 arrest observed between 12 and 24 h. Our data suggest that OTI-611, at optimal doses, forces cancer cells to exit the cell cycle, leading to programmed cell death, PARthanatos [6,7].

Another key highlight of this study is the cooperative interaction between OTI-611 and SOC therapies, such as etoposide, SN-38, and 5-FU. Etoposide is a topoisomerase II inhibitor anticancer agent that induces G2/M arrest [36]. Our real-time live cell cycle analysis data show that etoposide alone induces a dose-dependent accumulation of cells in G2/M phase; however, when combined with OTI-611, this effect is reprogrammed and both OTI-611 and etoposide act synergistically to induce G1 arrest in a dose-dependent manner. Furthermore, it suggests that CHD1L plays a key role in regulating the cell cycle via control of Cyclin D1. This mechanism is supported by OTI-611’s strong influence in downregulating Cyclin D1 expression regardless of etoposide dose. This aspect of OTI-611’s MOA is important because we have characterized for the first time that etoposide leads to the upregulation of Cyclin D1 expression in Colo678 cells. This off-target effect by etoposide may trigger MDR ‘s DNA damaging and apoptotic antitumor activity. This is a plausible conclusion based on our previous work that shows CHD1Li OTI-611 synergizes with chemotherapy and PARPi by reprogramming their cell death mechanism from apoptosis to PARthanatos, induced by OTI-611.

SN-38, the active metabolite of irinotecan and 5-FU SOC therapy in CRC [40], combined with OTI-611, enhanced G1 arrest, which amplified its effects and reduced tumor cell proliferation. 5-FU acts as an antimetabolite and exerts its anticancer activity by misincorporation into DNA and RNA and via thymidylate synthase inhibition [43]. When CHD1Li OTI-611 is combined with 5-FU, both drugs act cooperatively to induce G1 arrest in a dose-dependent manner. 5-FU combined with Irinotecan (FOLFIRI) is a SOC therapy for CRC [44]. Given the significant enhancement of antitumor activity when combined with SN-38 and 5-FU, introducing CHD1Li OTI-611 into FOLFIRI regimens may represent a promising future therapeutic strategy. In the context of targeted cancer therapy, OTI-611 combinations with Olaparib also blocked Olaparib’s G2/M arrest induction and reprogrammed cell cycle arrest to G1 phase in SUM149PT cells.

Taken together, CHD1Li OTI-611 demonstrates a strong ability to regulate the cell cycle by inducing sustained G1 arrest through Cyclin D1 downregulation. This dominant effect allows OTI-611 to override the MOA of other antitumor agents that typically cause G2/M arrest. Additionally, it reprograms these agents’ effects, enabling synergy with OTI-611 to further promote G1 arrest. Hence, future endeavors will focus on assessing the effect of OTI-611 on other oncogenic pathways with the goal of deepening our understanding on the pathways that CHD1L inhibition can modulate and to bring access to new therapeutic strategies that overcome MDR.

## 5. Conclusions

Our previous work has revealed and characterized the unique MOA of CHD1Li OTI-611, while also identifying new oncogenic functions of CHD1L [3,5,6,7]. OTI-611 functions as an allosteric inhibitor of CHD1L ATPase, trapping CHD1L on chromatin and thereby inhibiting its chromatin remodeling activity and other biological functions. For instance, CHD1L entrapment by OTI-611 leaves PAR chains in the nucleus unprotected, leading to the induction of PARthanatos, a form of programmed cell death [6,7]. This study further elucidates the mechanism by which OTI-611 promotes cell death, including its ability to arrest tumor cells in the G1 phase of the cell cycle. Notably, the most distinct feature of OTI-611’s MOA is its capacity to reprogram the mechanisms of other antitumor agents, enabling them to synergize with its own MOA. This synergy enhances DNA damage, reinforces G1 cell arrest, and amplifies the induction of PARthanatos. By doing so, this targeted combination strategy effectively bypasses MDR mechanisms, thereby inhibiting tumor progression and promoting cell death. The work herein further demonstrates the promise of OTI-611 as part of a targeted combination therapy to improve quality of life and survival for patients.

## Data Availability

The data that support the findings of this study are included in this published article and its Supplementary Material Files.

## Supplementary Materials

The following supporting information can be downloaded at https://zenodo.org/records/14908202, Figure S1: (A) Cell counts of SUM149PT BC cells when treated with OTI-611 or Etoposide. (B) Cell counts of SW620 CRC cells after treatment with OTI-611 or Etoposide. Data are shown as the mean of two independent experiments in triplicate technical replicates of two fields of view imaged every 2 h. Images were taken using the Incucyte S3 system at 10× magnification; Figure S2: (A) Cell cycle analysis of Colo678 cells treated with OTI-611 at 2.5 μM combined with Etoposide. (B) Cell cycle analysis of Colo678 cells treated with OTI-611 at 2.75 μM in combination with Etoposide. Data are shown as the mean of two independent experiments in triplicate technical replicates of two fields of view imaged every 2 h. Images were taken using the Incucyte S3 system at 10× magnification; Figure S3: (A) Cell cycle analysis of SUM149PT cells treated with OTI-611 at 1 μM combined with Etoposide. (B) Cell cycle analysis of SUM149PT cells treated with OTI-611 at 1.5 μM in combination with Etoposide. Data are shown as the mean of two independent experiments in triplicate technical replicates of two fields of view imaged every 2 h. Images were taken using the Incucyte S3 system at 10× magnification; Figure S4: (A) Cell cycle analysis of SW620 cells treated with OTI-611 at 2.5 μM combined with Etoposide. (B) Cell cycle analysis of SW620 cells treated with OTI-611 at 2.75 μM in combination with Etoposide. Data are shown as the mean of two independent experiments in triplicate technical replicates of two fields of view imaged every 2 h. Images were taken using the Incucyte S3 system at 10× magnification; Figure S5: (A) Cell count analysis of Colo678 cells treated with OTI-611 at 3 μM combined with Etoposide. (B) Cell count analysis of SUM149PT cells treated with OTI-611 at 2 μM in combination with Etoposide. (C) Cell count analysis of SW620 cells treated with OTI-611 at 3 μM in combination with Etoposide. Data are shown as the mean of two independent experiments in triplicate technical replicates of two fields of view imaged every 2 h. Images were taken using the Incucyte S3 system at 10× magnification; Figure S6: (A) Cell cycle analysis of Colo678 cells treated with OTI-611 at 2.5 μM combined with SN-38. (B) Cell cycle analysis of Colo678 cells treated with OTI-611 at 2.75 μM in combination with SN-38. Data are shown as the mean of two independent experiments in triplicate technical replicates of two fields of view imaged every 2 h. Images were taken using the Incucyte S3 system at 10× magnification; Figure S7: (A) Cell cycle analysis of Colo678 cells treated with OTI-611 at 2.5 μM combined with 5-FU. (B) Cell cycle analysis of Colo678 cells treated with OTI-611 at 2.75 μM in combination with 5-FU. Data are shown as the mean of two independent experiments in triplicate technical replicates of two fields of view imaged every 2 h. Images were taken using the Incucyte S3 system at 10× magnification; Figure S8: (A) Cell count analysis of Colo678 cells treated with OTI-611 at 3 μM combined with SN-38. (B) Cell count analysis of Colo678 cells treated with OTI-611 at 3 μM in combination with 5-FU. Data are shown as the mean of two independent experiments in triplicate technical replicates of two fields of view imaged every 2 h. Images were taken using the Incucyte S3 system at 10× magnification; Figure S9: (A) Cell cycle analysis of SUM149PT cells treated with OTI-611 at 0.5 μM combined with Olaparib. (B) Cell cycle analysis of SUM149PT cells treated with OTI-611 at 1.5 μM in combination with Olaparib. Data are shown as the mean of two independent experiments in triplicate technical replicates of two fields of view imaged every 2 h. Images were taken using the Incucyte S3 system at 10× magnification; Figure S10: (A) Cell count analysis of SUM149PT cells treated with Olaparib alone. (B) Cell count analysis of SUM149PT cells treated with OTI-611 at 0.5 μM in combination with Olaparib. (C) Cell count analysis of SUM149PT cells treated with OTI-611 at 1.5 μM in combination with Olaparib. (D) Cell count analysis of SUM149PT cells treated with OTI-611 at 2 μM in combination with Olaparib. Data are shown as the mean of two independent experiments in triplicate technical replicates of two fields of view imaged every 2 h. Images were taken using the Incucyte S3 system at 10× magnification; Video Sets S1–S10.
